# Dynein Heavy Chain 64C Differentially Regulates Cell Survival and Proliferation of Wingless-Producing Cells in *Drosophila melanogaster*

**DOI:** 10.3390/jdb9040043

**Published:** 2021-10-09

**Authors:** Ja-Young Kim, Orkhon Tsogtbaatar, Kyung-Ok Cho

**Affiliations:** 1 Department of Biological Sciences, Korea Advanced Institute of Science and Technology (KAIST), 291 Daehak-ro, Yuseong-gu, Daejeon 34141, Korea; jayoungk@cnu.ac.kr (J.-Y.K.); mandy54@kaist.ac.kr (O.T.); 2 Department of Medical Science, Chungnam National University, 266, Munhwa-ro, Jung-gu, Daejeon 35015, Korea

**Keywords:** Dhc64C, Dynein, Wg, Sona, wing disc, apoptosis, microtubule motors

## Abstract

Dynein is a multi-subunit motor protein that moves toward the minus-end of microtubules, and plays important roles in fly development. We identified *Dhc64C^m115^*, a new mutant allele of the fly *Dynein heavy chain 64C* (*Dhc64C*) gene whose heterozygotes survive against lethality induced by overexpression of Sol narae (Sona). Sona is a secreted metalloprotease that positively regulates Wingless (Wg) signaling, and promotes cell survival and proliferation. Knockdown of *Dhc64C* in fly wings induced extensive cell death accompanied by widespread and disorganized expression of Wg. The disrupted pattern of the Wg protein was due to cell death of the Wg-producing cells at the DV midline and overproliferation of the Wg-producing cells at the hinge in disorganized ways. Coexpression of *Dhc64C RNAi* and *p35* resulted in no cell death and normal pattern of Wg, demonstrating that cell death is responsible for all phenotypes induced by *Dhc64C RNAi* expression. The effect of Dhc64C on Wg-producing cells was unique among components of Dynein and other microtubule motors. We propose that Dhc64C differentially regulates survival of Wg-producing cells, which is essential for maintaining normal expression pattern of Wg for wing development.

## 1. Introduction

Apoptosis is a programmed cell death critical for the development and homeostasis of all organisms, which functions by eliminating unwanted cells generated under both normal and stress conditions [1]. Apoptosis often occurs concomitantly with compensatory proliferation to maintain tissue size and shape [2]. *Drosophila melanogaster* is a great model system for studying apoptosis and compensatory proliferation whose mechanism and components are highly conserved during evolution [3,4,5]. The wing imaginal disc, the primordium of the adult wing, is especially suitable for studying these processes because it shows little apoptosis during normal development but develops to a normal wing even with 40–60% of apoptotic cells via efficient compensatory proliferation [6].

The family of A Disintegrin and Metalloproteinase with ThromboSpondin motifs (ADAMTS) has important roles in cell proliferation, cell survival, cell migration, blood coagulation, angiogenesis in mammals, and their malfunctions result in numerous diseases including cancer, arthrosclerosis, and arthritis [7,8,9,10]. Sol narae (Sona) is a member of fly ADAMTS proteins, and positively regulates Wg signaling by promoting secretion of Wg [11]. Sona is also important for cell survival and compensatory proliferation by increasing the level of Cyclin D for cell division in order to maintain tissue size and shape [11,12].

To understand the function of Sona, our laboratory previously carried out a genetic screen, and identified suppressors that overcome lethality induced by overexpressed Sona. Several suppressors that have mutations in *wntless*, *pou domain factor 3 (pdm3)*, *archipelago* (*ago*), *arrow* (*arr*) and *anastral spindle 3* genes have been reported [11,13,14,15,16]. The focus of this study is *m115*, one of the suppressors that has a missense mutation in the *Dhc64C* gene. Fly Dhc64C is a component of cytoplasmic Dynein, a microtubule force-producing protein motor moving toward the minus end of microtubules essential for organelle transport and centrosome assembly [16]. Cytoplasmic Dynein is a macromolecular complex that contains two heavy, two intermediate, two light intermediate and several light chains. The two identical heavy chains in Dynein form dimers and have motor machinery that transduces chemical energy derived from ATP hydrolysis into mechanical force [17,18].

We report here that *sona* and *Dhc64C* show a positive genetic interaction, and the knockdown of Dhc64C induces apoptosis of Wg-producing cells in the DV midline, whereas uncontrolled compensatory proliferation of Wg-producing cells originated from the hinge region, which resulted in abnormally high numbers of Wg-producing cells in entire wing discs. Knockdown of genes encoding other subunits in Dynein or Kinesin did not show such phenotypes, suggesting that Dhc64C plays a unique role in cell survival and proliferation of Wg-producing cells in fly wing discs.

## 2. Materials and Methods

### 2.1. Fly Strains

*Gal4* drivers such as *patched* (*ptc*)-*Gal4*, *30A-Gal4*, *nubbin* (*nub*)-*Gal4*, *engrailed* (*en*)-*Gal4*, *C96-gal4*, *UAS* lines such as *UAS-GFP*, *UAS-Dhc64C RNAi* (BL28749), *UAS-Khc RNAi* (BL35770), and flies for mapping were obtained from Bloomington Stock Center. *UAS-cut up RNAi* (v43115), *UAS-Klp64D RNAi* (NIG10642R-1) and *UAS-Dhc64C RNAi* (NIG7507R-2) were obtained from Vienna Drosophila Resource Center and National Institute of Genetics. Deficiency lines (*Df(3L)BSC436*, *Df(3L)ED210*, *Df(3L)Exel6102*) that uncover the *Dhc64C* gene and, *Dhc64C^6.6−16^* (BL32015) were used for complementation test of *Dhc64C*. We used *UAS-Sona* that had been generated in previous study [11]. Fly cultures were carried out at 25 °C unless indicated otherwise.

### 2.2. Immunohistochemistry

Wing discs of the third instar larvae were dissected, fixed, blocked, and incubated with the primary antibodies, as described [19]. After washing several times, samples were incubated with secondary antibodies in washing buffer for 2 h at room temperature and stained with 4′, 6-diamidino-2-phenylindole (DAPI). Then, samples were mounted using Vectashield (Vector Laboratories, Burlingame, CA, USA). All confocal images were acquired using Zeiss LSM710 confocal microscope and ZEN software.

Primary antibodies were used in the following dilutions: chicken anti-β-gal (ab 9361), 1:100; rabbit anti-cleaved Caspase-3 (Cell Signaling Technology, Danvers, MA, USA), 1:250; rabbit anti-Dlg, 1:500 [20], mouse anti-Wg (DSHB #4D4), 1:100; mouse anti-Cut (DSHB #2B10), 1:100; guinea pig anti-Senseless (gift from Hugo Bellen), 1:1000 sheep anti-GFP (Ab direct serotec), 1:100. Secondary antibodies conjugated with Cy3 (1:200), Cy5 (1:500) and FITC (1:200) were from Alexa Flour (Jackson Immunoresearches, West Grove, PA, USA).

### 2.3. Adult Wing Mounting

The left wings of 2–7 days old flies were dissected and mounted in Gary’s magic mounting solution (Canada Balsam and methyl salicylate, 4:1). The wing images were obtained using Zeiss Axio imager M2 and Axiocam software. All wing images were taken with 50× magnification.

## 3. Results

### 3.1. A Sona Suppressor m115 has a Mutation in the ATPases Associated with Various Cellular Activities 3 (AAA3) Domain of Dhc64C Protein

**Figure 1:**
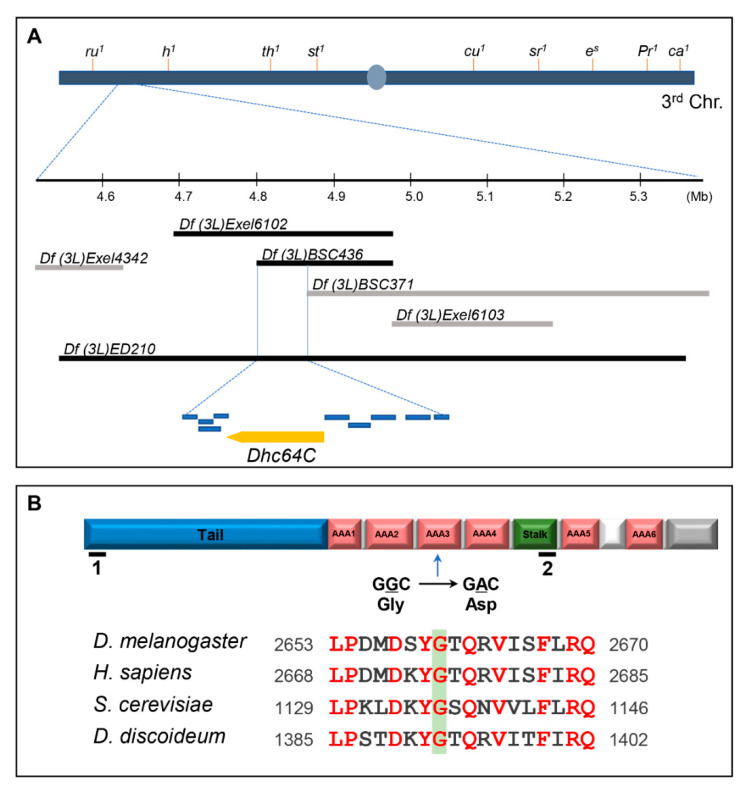
The lethal site in the *sona* suppressor *m115* is mapped to the *Dhc64C* gene. (**A**) Mapping of the lethal site in *m115* by meiotic and deficiency mapping. Meiotic mapping localized the lethal site in *m115* between *ru* and *h*, which is further explained in Table S1. Multiple deficiencies were used to narrow it down to 64B17-64C1. Deleted range in the two deficiencies that uncover the lethal site of *m115* are marked with black bars. Other deficiencies are marked with gray bars. *Dhc64C* and nine genes in the region common in the two deficiencies are presented as yellow and blue bars, respectively. Their names are listed in Table S2. (**B**) The domain structure of Dynein heavy chain. A point mutation from G to A results in one amino acid change from glycine (G) to aspartic acid (D) in the AAA3 domain of *Dhc64C^m115^*. The region including the mutated site of *Dhc64C^m115^* is conserved in Dhc proteins of model organisms. Red letters indicate amino acid residues conserved in these organisms. RNAi target site for BL28749 and NIG7507R-2 are marked by black lines with number 1 and 2, respectively.

Heterozygous *m115* was identified in a genetic screen as one of the 28 suppressors that overcome the lethality induced by Sona overexpression [11,14,16]. Homozygous *m115* was embryonic lethal, so we mapped the lethal site by meiotic and deficiency mapping under the assumption that the lethal mutation of the *m115* is responsible for suppression of Sona-induced lethality in *m115* heterozygotes. Meiotic mapping localized the lethal site between the two markers in the third chromosome, *roughoid* (61F8) and *hairy* (66D10) (Supplementary Table S1). Subsequent deficiency mapping narrowed down the lethal site between 64B17 and 64C1, in which ten genes are present (Figure 1A). To identify the gene that harbors the lethal site, we then carried out complementation tests. One of the mutants that did not complement the lethality of *m115* was *Dhc64C^6.6−16^*, a hypomorph that can develop to adulthood (Supplementary Table S2).

Genomic sequencing of *Dhc64C^m115^* flies identified a guanine to adenosine transition that changes glycine to aspartic acid in the AAA3 domain of the *Dhc64C* protein, which is consistent with the mechanism of the mutagen ethyl methanesulfonate (EMS) used in our genetic screen, guanine alkylation, which changes GC pairs to AT pairs [11,14]. This glycine residue is conserved in all cytoplasmic Dynein heavy chain proteins examined (Figure 1B). The ATPase activity of AAA3 domain is especially important for release of Dynein from microtubules for the subsequent movement of Dynein along the microtubules [21]. *Dhc64C* is most homologous to the human cytoplasmic Dynein 1 heavy chain 1 (5NUG_A) with 72% identity and 85% similarity. It also shows 72% and 57% similarity to Dhc of *D. discoideum* and *S. cerevisiae*, respectively.

**Figure 2:**
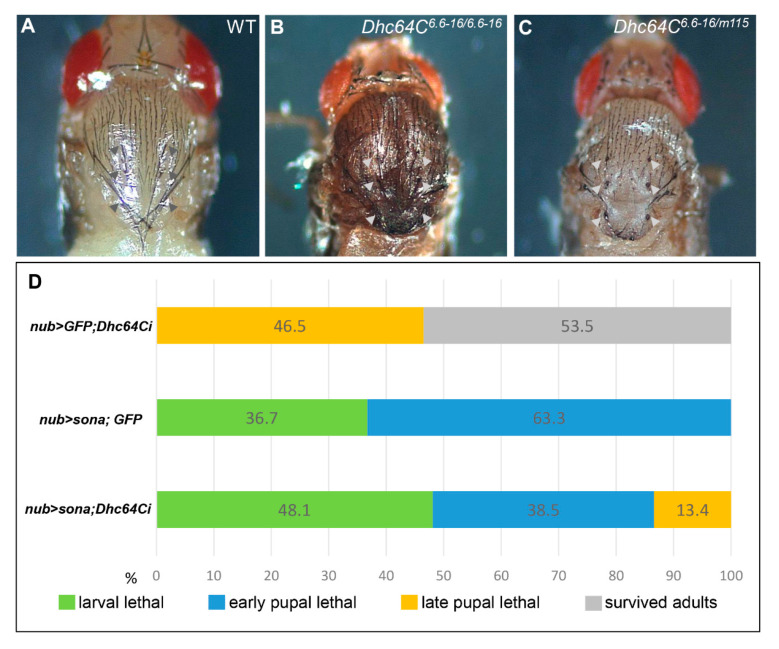
*Dhc64C* has a positive genetic interaction with *sona*. (**A–C**) Scutellar bristles are defective in *Dhc64C* mutants. The normal scutellum of wild type *Canton-S* (*CS*) pupae ((**A**), arrowheads). Scutellar bristles are short and disrupted in hypomorphic *Dhc64C^6.6−16/6.6−16^* adults ((**B**), arrowheads) and transheterozygous *Dhc64C^6.6−16^*/*Dhc64C^m115^* pharate adults ((**C**), arrowheads). (**D**) Knockdown of Dhc64C partially rescues Sona-induced lethality. Fly genotypes and developmental states in percentages are shown.

As mentioned above, *Dhc64C^6.6−16^* can develop to adults, but *m115/Dhc64C^6.6−16^* only survived up pharate adults, indicating that the mutation in *m115* is responsible for the lethality of the heterozygotes. Pupae of both *m115/Dhc64C^6.6−16^* transheterozygotes and *Dhc64C^6.6−16^* homozygotes had short and deformed scutellar bristles, also indicating that bristle phenotype of *Dhc64C^6.6−16^* is not complemented by *m115* (Figure 2A–C). This bristle phenotype is consistent with the importance of Dhc64C in bristle formation [22]. In sum, both lethality and bristle defects are due to the mutation in the *Dhc64C* gene of *m115*, so *m115* was named *Dhc64C^m115^* as a new *Dhc64C* allele.

### 3.2. Knockdown of Dhc64C Reduces the Severity of Sona-Induced Lethality

We then examined whether the reduction in the level of wild-type Dhc64C by *Dhc64C RNAi* expression is as efficient as Dhc64C^m115^ protein in suppressing Sona-induced lethality. For this work, we tested two *Dhc64C RNAi* (*Dhc64Ci*) lines, BL28749 and VDRC 7507R-2, covering different parts of the *Dhc64C* transcript (Figure 1B-1,2). Their expression by crossing with various *Gal4* lines at 25 °C resulted in embryonic to late pupal lethality with small-sized larvae (Supplementary Table S3). Since both *RNAi* lines showed similar phenotypes, BL28749 was chosen for the knockdown of Dhc64C study in this report.

We generated *UAS-GFP; UAS-Dhc64Ci*, *UAS-sona; UAS-GFP*, and *UAS-sona; UAS-Dhc64Ci* flies, crossed them with *nub-Gal4*, and compared the lethal rate of their progenies at 18 °C (Figure 2D). All *nub > sona*; *GFP* flies (*n* = 109) were larval or early pupal lethal, while 46.5% of *nub-Gal4 > UAS-GFP; UAS-Dhc64Ci* (*nub > GFP; Dhc64Ci*) flies (*n* = 43) were late pupal lethal. In contrast, 13.4% of *nub > sona; Dhc64Ci* flies (*n* = 52) could develop up to late pupal stage. Therefore, the knockdown of Dhc64C reduced the severity of Sona-induced lethality, though less efficiently than the *Dhc64C^m115^* allele. These results are consistent with the identification of *Dhc64C^m115^* as a *sona* suppressor in the aforementioned genetic screen.

### 3.3. Knockdown of Dhc64C Induces Apoptosis

**Figure 3:**
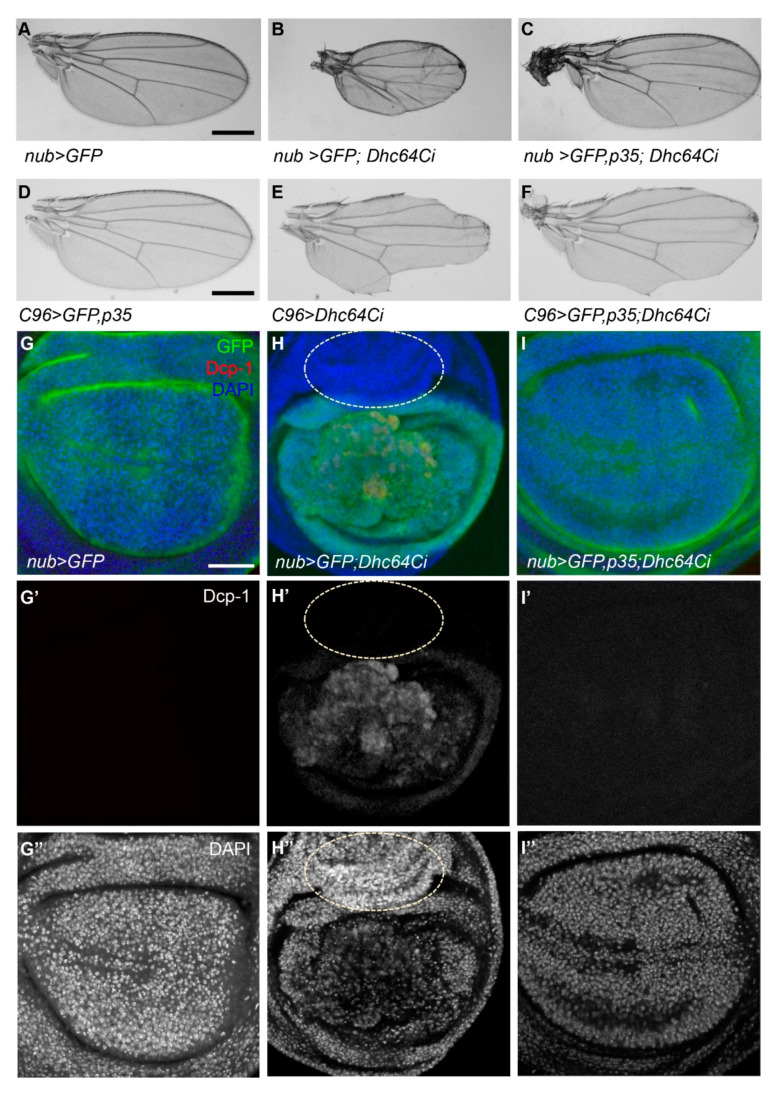
The knockdown of Dhc64C induces apoptosis. (**A**–**C**) Knockdown of Dhc64C induces small wing phenotype, which can be rescued by coexpression of p35. Compared to control *nub-Gal4/+* wings (**A**), *nub > GFP; Dhc64Ci* wings from rare survivors cultured at 18 °C are small and wrinkled (**B**), and *nub > GFP, p35; Dhc64Ci* wings are normal (**C**). Scale bar 500 µm. *n* = 10 each. (**D**–**F**) Compared to the normal *C96 > GFP, p35* wings (**D**), *C96 > Dhc64Ci* wings are severely notched (**E**). This notching phenotype of C96 > GFP; Dhc64Ci wings is noticeably rescued by p35 (**F**). Scale bar, 500 µm. *n* = 10 each (**G**–**I**) Knockdown of Dhc64C in the wing pouch region induces both apoptosis and cell proliferation, which are rescued by p35. Activated Dcp1 is detected in wing discs of the *nub > GFP; Dhc64Ci* flies (**H’**), which are not detected from *nub > GFP* and *nub > GFP, p35; Dhc64Ci* (**G’**,**I’**, respectively) DAPI staining shows overall organization of wing pouch cells (**G”**–**I”**). All apoptosis-related phenomena are rescued by coexpression of p35 (**I**). Abnormal growth of hinge region outside of *nub-Gal4*-expressing region is marked with dotted circles (**H**–**H’’**). Anterior to the left. Scale bar 100 µm.

Since the knockdown of Sona induces cell death and generates small wing phenotype [11,12], we checked whether the knockdown of Dhc64C also generates small wings using multiple *Gal4* lines. Some *nub > Dhc64Ci* flies at 18 °C developed into adults as described (Figure 2D), and all their wings were either absent or smaller than control *nub > GFP* wings (*n* > 20, each) (Figure 3A,B). With a similar tendency, all *C96 > Dhc64Ci* flies (*n* = 12) had notched wings (Figure 3D,E). All *ptc > GFP; Dhc64Ci* wings (*n* > 20) were not flat with some abnormally grown structures, their wing size was also smaller than wild-type wings, and the region between L3 and L4 veins was narrower than wild-type wings at 22 °C (Supplementary Figure S1A,B). Therefore, knockdown of Dhc64C by multiple *Gal4* lines results in small or notched wings.

We then tested whether apoptosis induced by the knockdown of Dhc64C is responsible for these small or notched wing phenotypes. Indeed, co-expression of *Dhc64C RNAi* and *p35* restored wing size of *nub > Dhc64Ci* and reduced notching phenotype of *C96 > Dhc64Ci* flies (Figure 3C,F). Consistent with these adult wing phenotypes, *nub > GFP; Dhc64Ci* wing discs had activated Drosophila caspase 1 (Dcp-1) signal in the GFP-expressing region, while control *nub > GFP* and *nub > GFP, p35; Dhc64Ci* wing discs had no Dcp-1 signal in all wing discs examined (Figure 3G’–I’). Furthermore, p35 completely normalized overall organization of *nub > GFP, p35; Dhc64Ci* wing discs compared to *nub > GFP; Dhc64Ci* wing discs (Figure 3G’’–I’’). These demonstrate that the knockdown of Dhc64C induces apoptosis.

Notched wing phenotype of *C96 > Dhc64Ci* flies may also be due to cell death, specifically at the DV midline region (Figure 3D–F). We therefore checked whether Senseless (Sens), a transcription factor involved in the differentiation of wing margin cells into sensory organs, is affected in *C96 > Dhc64Ci* flies [23,24]. Sens was not properly expressed at the DV midline in *C96 > Dhc64Ci* wings compared to control wings (Supplementary Figure S2A,B). Co-expression of *Dhc64C RNAi* with p35 partially restored Sens expression (Supplementary Figure S2C). In sum, apoptosis by knockdown of Dhc64C is responsible for small or notched wing phenotypes.

### 3.4. Knockdown of Dhc64C Induces Overproliferation in a Non-Cell Autonomous Manner

We have shown above that GFP-positive (GFP^+^) cells in *nub > GFP; Dhc64Ci* discs exhibit cell death because of *Dhc64 RNAi* expression driven by the *nub-Gal4* driver, whereas GFP-negative (GFP^−^) cells in these discs without any *Dhc64 RNAi* expression showed no sign of cell death (Figure 3H). Interestingly, some GFP^−^ cells in the region of outer ring hinge showed overproliferation and appeared to move into the wing pouch region where GFP^+^ cells are present (Figure 3H–H”). Obviously, these overproliferated GFP^−^ cells did not express apoptotic marker DCP-1 because *Dhc64C RNAi* is not expressed in these cells (dotted circle in Figure 3H–H’’). In another *nub > GFP; Dhc64Ci* disc, these GFP^−^ cells were present in between GFP^+^ cells probably by moving into the pouch from the hinge region (red arrows in Supplementary Figure S1C). This phenomenon implied that cell death by the knockdown of *Dhc64C* induces overproliferation of neighboring cells in a non-cell autonomous manner.

When p35 was coexpressed with *Dhc64C RNAi* in *nub > GFP, p35; Dhc64Ci* wing discs, neither cell death of GFP^+^ cells nor overproliferation of GFP^-^ cells were detected (Figure 3I–I’’). Therefore, we concluded that the knockdown of Dhc64C induces cell death in a cell-autonomous manner and then overproliferation of neighboring cells in a non-cell-autonomous manner.

### 3.5. The Knockdown of Dhc64C Increases the Level of Wg in the Entire Wing Pouch

Co-occurrence of apoptosis and cell proliferation by the knockdown of Dhc64C was an intriguing phenomenon, so we examined whether the knockdown of Dhc64C affects the expression level or pattern of Wg based on the role of Sona in Wg signaling [11,25]. Wg signaling is known to be involved in compensatory proliferation and hyperplastic overgrowth caused by apoptotic cells [26].

**Figure 4:**
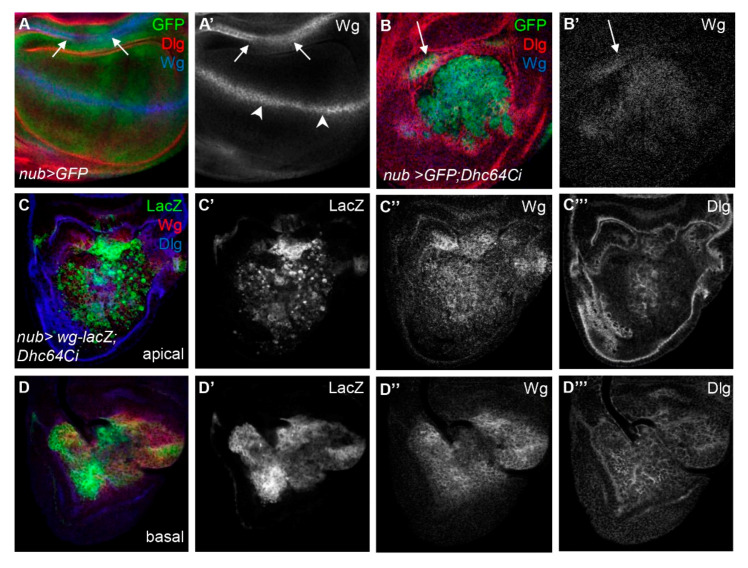
Knockdown of Dhc64C disrupts expression pattern of Wg. (**A**–**A’**,**B**–**B’**) Knockdown of Dhc64C results in broad expression of Wg in the wing pouch. Pattern of Wg protein at the DV midline (arrowheads) and in the inner ring (arrows) in control wing disc (**A**,**A’**) and extensively disorganized and widely distributed pattern of Wg in severely affected *nub > GFP*; Dhc64Ci wing discs (**B**,**B’**). (**C**–**C’’**,**D**–**D’’**) The broad expression of Wg protein is due to widespread Wg-producing cells in both apical (**C**–**C’’**) and basal (**D**–**D’’**) regions of *nub > GFP; Dhc64Ci* wing discs.The expression pattern of the Dlg is also disrupted both in apical (**C’’’**) and basal (**D’’’**).

We therefore examined the pattern of Wg protein in *nub > GFP; Dhc64Ci* wing discs. We found that Wg protein was present in the entire pouch of *nub > GFP; Dhc64Ci* wing discs, unlike the control *nub > GFP* wing discs in which Wg was present at the DV midline and the inner ring hinge (Figure 4A,B). To check whether the change in Wg pattern by the knockdown of Dhc64C is a phenotype common to other components in microtubule motors, we knocked down the level of *cut up* (*ctp*), which encodes a light chain of cytoplasmic Dynein [27,28], *Kinesin heavy chain* (*Khc*), which encodes the heavy chain of Kinesin-1 [29,30], and *Kinesin-like protein at 64D* (*Klp64D*), which encodes a motor subunit of the Kinesin-2 [31,32] (Supplementary Figure S3). None of them showed a Wg pattern similar to that of Dhc64C knockdown, suggesting that Dhc64C is unique among microtubule motor subunits whose loss results in overall increase in Wg pattern in the entire pouch.

### 3.6. Knockdown of Dhc64C Induces Cell Death of Wg-Expressing Cells in the Apical Region

We then examined how the pattern of Wg is changed by the knockdown of Dhc64C. The pattern of Wg protein was similar to that of Wg-lacZ cells in both apical and basal regions of *nub > wg-lacZ; Dhc64Ci* wing discs, demonstrating that the widespread Wg-expressing cells are responsible for the broad distribution of Wg protein (Figure 4C,D). One noticeable difference between Wg-lacZ cells the apical and basal regions was the spottiness of the Wg-lacZ cells in the apical but not the basal region (Figure 4C’,D’).

**Figure 5:**
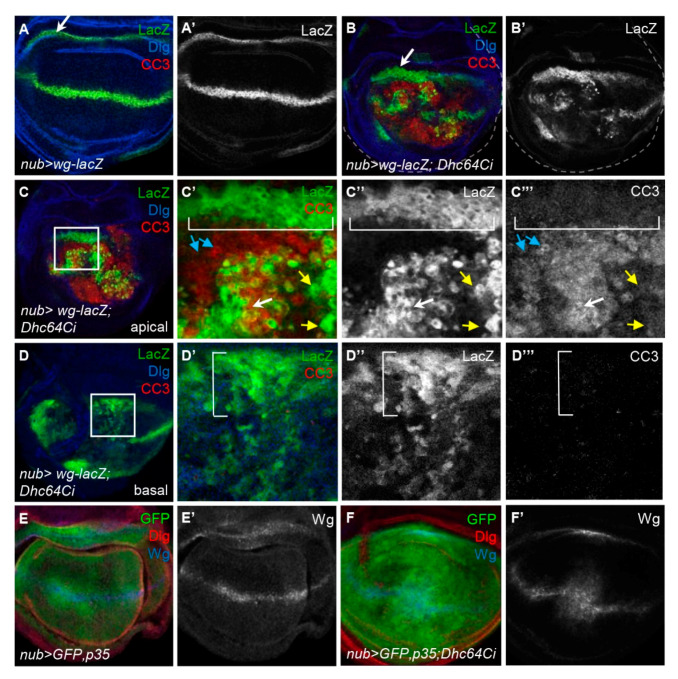
Knockdown of Dhc64C induces cell death and cell proliferation of Wg-producing cells in the apical and basal region of wing discs, respectively. (**A**–**A’**,**B**–**B’**) Comparison of Wg-lacZ pattern between a *nub-Gal4 > wg-lacZ* control disc (**A**,**A’**) and a *nub-Gal4 > wg-lacZ; Dhc64Ci* wing disc (**B**,**B’**) at the basolateral region. (**B**) is one of Z-stack images of a *nub-Gal4 > wg-lacZ; Dhc64Ci* wing disc in Figure S4. (**C**,**D**) Apoptotic cells are present in the apical region but not in the basal region. Images representing apical (**C**) and basal (**D**) region of a *nub-Gal4 > wg-lacZ; Dhc64Ci* wing disc are selected from the same Z-stack images in Figure S4. The boxed regions in **C** and **D** are magnified in **C’**–**C’’’** and **D’**–**D’’’**. Apically located Wg-lacZ cells in the hinge region (brackets) show no apoptosis (**C**). Some Wg-lacZ cells (white arrows) are apoptotic while others (yellow arrows) are not in the pouch (**C**). Most pouch cells that do not express Wg-lacZ (blue arrows) are apoptotic (**C**). Basally located Wg-lacZ cells in the hinge region are increased in their number, and were dispersed into the pouch region (brackets in **D’**–**D’’’**). (**E**,**E’**,**F**,**F’**) Abnormal Wg pattern induced by the knockdown of Dhc64C is rescued by p35. Compared to Wg pattern of *nub > GFP*, p35 wing discs (**E**), that of *nub > GFP; p35, Dhc64Ci* wing (**F**) are almost normal unlike Figure 4B.

The spotty Wg-lacZ cells in the apical region of *nub > wg-lacZ; Dhc64Ci* wing discs indeed had the cleaved Caspase 3 (CC3), a marker for apoptotic cells [30] (Figure 5A,B). Consistent with this result, apoptosis was most severe in the apical region, gradually decreased toward the basal region, and was almost absent in the basal region (Supplementary Figure S4). This suggests that the apically located cells are more prone to cell death by the knockdown of Dhc64C.

### 3.7. Knockdown of Dhc64C Does Not Induce Cell Death but Induces Overproliferation of Wg-Expressing Cells in the Inner Hinge

We further analyzed the regional difference in apoptosis of Wg-lacZ cells of *nub > wg-lacZ; Dhc64Ci* wing discs. In the apical region, most Wg-lacZ cells near the DV midline, as well as surrounding pouch cells, contained CC3 (white and blue arrows in Figure 5C’–C”’). However, the Wg-lacZ cells in the inner hinge did not have CC3 (brackets in Figure 5C’–C”’) and even became somewhat thicker (arrows in Figure 5A,B) although *Dhc64C RNAi* in that region is also driven by *nub-Gal4*. Similarly, the Wg-lacZ cells in the inner hinge of the basal region had no CC3 signal but instead were noticeably increased in number and dispersed into the pouch (bracket in Figure 5D; Supplementary Figure S4). Therefore, the Wg-lacZ cells in the inner hinge were especially resistant to apoptosis, unlike those in the pouch. The movement of these hinge cells toward the wing pouch has been also reported when the wing pouch is damaged by cell death [33,34].

Consistent with the phenotypic rescue of *nub > Dhc64Ci* flies by the coexpression of p35 (Figure 3C,I), p35 also rescued the distorted pattern of Wg induced by the knockdown of Dhc64C (Figure 5E,F). Therefore, cell death induced by the knockdown of Dhc64C is responsible for all phenotypes described so far, including overproliferation of Wg-lacZ cells in the inner hinge.

## 4. Discussion

We report here that *Dhc64C^m115^* as a heterozygote is isolated as a suppressor of Sona-induced lethality, and *Dhc64C* and *sona* have a positive genetic interaction (Figure 1 and Figure 2). The mutated site in Dhc64C^m115^ protein is 5 amino acid residues away from the ATPase site in the AAA3 domain, and this mutation may compromise the MT release by reducing or abolishing the ATPase activity of the AAA3 domain [21]. Dhc64C was especially important for cell survival of the apically located Wg-producing cells in wing discs (Figure 4). Interestingly, cell death caused by knockdown of Dhc64C induced overproliferation of basally located Wg-producing cells and widespread expression of Wg in the wing pouch. These suggest that Dhc64C is required for cell survival and proper compensatory proliferation for maintenance of tissue size and shape. These properties of Dhc64C were not observed in other Dynein and Kinesin components, indicating that Dhc64C plays a unique role that is not shared by other motor components. Sona is also important for cell survival and cell proliferation, so Dhc64C and Sona may cooperate to carry out these functions.

We have shown that several *sona* suppressors function as dimers [13,14,15], and Arr^m7^ mutant protein acts as a dominant negative *sona* suppressor [13]. Dynein complex contains a homodimer of Dhc, so Dhc64C^m115^-Dhc64C dimer in *Dhc64C^m115^/+* heterozygotes may act as a dominant negative and compromise the activity of Dynein but the amount of Dhc64C-Dhc64C dimer in *Dhc64C^m115^/+* heterozygotes may still be enough to support normal development. This explains how *Dhc64C^m115^/+* heterozygotes develop normally to adults, and yet suppress Sona-induced lethality. This may also explain why Sona is more efficiently suppressed in *Dhc64C^m115^/+* heterozygotes than by the expression of *Dhc64C RNAi*. Knockdown of Dhc64C by *Dhc64C RNAi* decreases the amount of wild-type Dhc64C, but not to the right amount for suppressing the excess amount of Sona. In addition, ectopic expression of *Dhc64C RNAi* by *Gal4* drivers such as *nub-Gal4* may not be adequate to ensure proper fly development. Further studies to compare the ability of Sona suppression of *Dhc64C^m115^/+* heterozygotes and *Dhc64C RNAi* are required. Whether Sona and Dhc64C directly interact is another interesting question to explore.

The knockdown of Dhc64C induces apoptosis, overproliferation, overall increase in the level of Wg in wing discs, and the formation of small adult wings (Figure 3 and Figure 4). These phenotypes are also induced by the knockdown of Rab5 [35]. Rab5 is involved in maturation of early endosomes to late endosomes such as multivesicular bodies (MVB) [36,37]. Sona colocalizes with Rab5 in early endosomes and is then released via exosomes to extracellular space by fusion of MVB to plasma membrane [11]. Since Rab5-containing early endosomes are transported by cytoplasmic Dynein, both Rab5 and Dynein are essential for the secretion of exosomal Sona [36,38]. Whether the knockdowns of other components in endosomal pathway show both cell death and overproliferation phenotypes is an interesting question that has yet to be explored.

Cytoplasmic Dynein helps to position Golgi complex and other organelles in the cell and transports membranous organelles such as Golgi-derived membranes, endosomes and lysosomes [39]. Interestingly, Sona and Wg are secreted both by Golgi transport and via exosomes that are derived from the endosomal pathway [11,40]. Given that the Dhc64C^m115^ mutant is identified as a *sona* suppressor, Dhc64C may play a role in transporting Sona, and thereby affects Wg signaling. Further analysis is necessary to find out the biochemical relationship between Dhc64C, Sona and Wg.

One important finding is that the knockdown of Dhc64C causes cell death of the Wg-expressing cells in the apical region, while causing cell proliferation of the Wg-expressing cells in the basal region of wing discs (Figure 4 and Figure 5). Since hinge cells also proliferate and move to the pouch region when pouch cells become apoptotic under stress conditions [33,34], these Wg-lacZ cells in the basal region are most likely originated from the hinge, moving to the basal region of wing discs to repair the site emptied by cell death. Therefore, the apically located Wg-lacZ cells are the original residents of the disc proper of the wing pouch, while the basally located Wg-lacZ cells are the descendants of the hinge cells originated from peripodial epithelium. It is essential to understand how Dhc64C and Sona function in these hinge cells for cell survival and proliferation.

## 5. Conclusions

We identified *Dhc64C^m115^* as a suppressor of Sona-induced lethality. Dhc64C is important for cell survival, especially apical Wg-producing cells in wing discs. Therefore, Dhc64C is involved in Wg signaling by apical transport of *wg* transcript, and also in cell survival of Wg-producing cells. Since Sona is also important for cell survival, we propose that Dhc64C and Sona cooperate to promote cell survival by positively regulating Wg signaling.

## Data Availability

The materials described in the manuscript, including all relevant raw data, will be freely available to any researcher wishing to use them for non-commercial purposes, without breaching participant confidentiality. The datasets used or analyzed during the current study are available from the corresponding author on reasonable request.

## Supplementary Materials

The following are available online at www.mdpi.com/article/10.3390/jdb9040043/s1, Table S1: Meiotic mapping of the *m115* suppressor; Table S2: Ten potential genes for *m115* identified by deficiency mapping; Table S3: The knockdown phenotypes of the two *Dhc64C RNAi* lines are identical; Figure S1: Dhc64C is important for cell survival; Figure S2: Knockdown of Dhc64C reduces the expression level of Sens, which is partially rescued by coexpression of p35; Figure S3: Subunits in Dynein and Kinesin microtubule motors do not induce overproliferation of Wg-producing cells; Figure S4: The Wg-lacZ cells in the apical region are apoptotic unlike the Wg-lacZ cells in the basal region.
