# Supplementary material for: Dynein Heavy Chain 64C Differentially Regulates Cell Survival and Proliferation of Wingless-Producing Cells in Drosophila melanogaster
Source: J Dev Biol. 2021 Oct 9;9(4):43. doi: 10.3390/jdb9040043 (PMC8544498; doi:10.3390/jdb9040043)
Supplement: Supplementary file 1 [file jdb-09-00043-s001.zip › jdb-1308015-supplementary.pdf]

Table S1. (Kim and Orkhon et al)

A

recombinants								lethality
+	+	th^1	st^1	cu^1	+	+	+	+
+	+	th^1	st^1	cu^1	sr^1	e^s	+	+
+	+	+	+	+	sr^1	e^s	ca^1	+
+	+	+	+	cu^1	sr^1	e^s	ca^1	+
+	+	+	+	+	+	e^s	ca^1	+
+	+	+	st^1	cu^1	sr^1	+	+	+
+	+	th^1	st^1	cu^1	sr^1	e^s	+	+
+	+	th^1	st^1	+	+	+	+	+
+	h^1	+	+	+	sr^1	e^s	ca^1	-
+	h^1	th^1	st^1	cu^1	sr^1	e^s	+	-
+	h^1	th^1	st^1	cu^1	sr^1	+	+	-
+	h^1	th^1	st^1	cu^1	+	+	+	-
+	h^1	+	+	+	+	+	+	-
+	h^1	+	+	+	+	+	ca^1	-

B

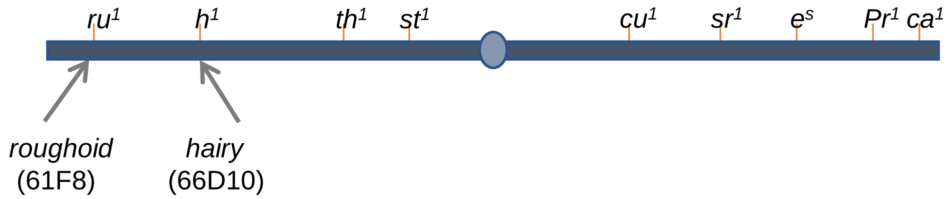

Table S1. Meiotic mapping of the *m115* suppressor

(A, B) multiple recombinants were obtained by crossing *m115* and #576 yielded, and only recombinants that has crossover between *roughoid* (*ru*) and *hairy (h)* () contained the lethal site (boxed in A). Therefore, the lethal site of *m115* was mapped in between 64F8-66D10 (B).

Table S2. (Kim and Orkhon et al)

Cytology	Symbol
64B17-64B17	CR45821
64B17-64B17	CR45740
64B17-64B17	CR44528
64B17-64B17	CG7509
64B17-64B17	CG18808
64B17-64C1	Dhc64C
64C1-64C1	Aats-leu
64C1-64C1	CG13708
64C1-64C1	CG13707
64C1-64C1	CG32237

Table S2. Ten potential genes for *m115* identified by deficiency mapping
Deficiency mapping (Fig. 1A) narrowed down the lethal site to 64B17-64C1, and ten genes present in this region are listed. Among these genes, complementation tests showed that *m115* has a lethal mutation in the *Dhc64C* gene.

Table S3. (Kim and Orkhon et al)

***Gal4* drivers**	***UAS-Dhc64C RNAi* (2nd chr) (NIG #7507R-2)**	***UAS-Dhc64C RNAi* (3rd chr) (BDSC #25791)**
nub-Gal4	small larvae, early pupal lethal	small larvae, early pupal lethal
ey-Gal4	embryo to pupal lethal	small larvae, late pupal lethal
en-Gal4	embryo to 3rd instar larval lethal	embryo to 1st instar larval lethal
ptc-Gal4	embryo to early pupal lethal	early pupal lethal
30A-Gal4	larval to pupal lethal	pupal lethal

Table S3. The knockdown phenotypes of the two *Dhc64C RNAi* lines are identical

Both NIG #7507R-2 and BDSC #28749 lines whose *Dhc64C RNAi* transgenes are inserted in the second and the third chromosome, respectively, induce similar phenotypes at 25°C. Their binding sites in *Dhc64C* transcripts are marked in Fig. 1B.

Fig S1. (Kim and Orkhon et al)

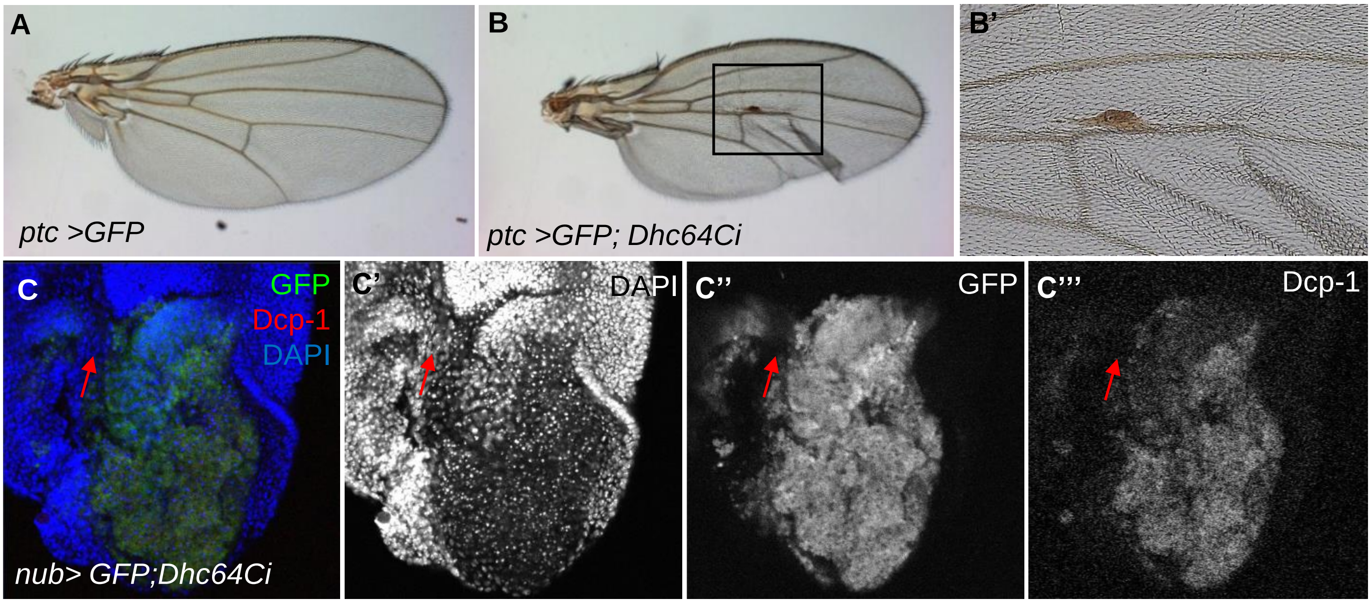

Figure S1. Dhc64C is important for cell survival

(A, B) Knockdown of *Dhc64C* by *ptc-Gal4* at 22℃ resulted in uneven growth and reduction of the wing area between L3 and L4 veins (E, black box). High magnification in (B') shows defects in tissue polarity.

(C) Knockdown of *Dhc64C* in wing discs by *nub-Gal4* at 25℃ resulted in abnormal morphology and cell death detected by *Drosophila* caspase-1 (Dcp-1). Red arrows indicate some GFP negative cells in between wing pouch and hinge regions that are not apoptotic.

Fig S2. (Kim and Orkhon et al)

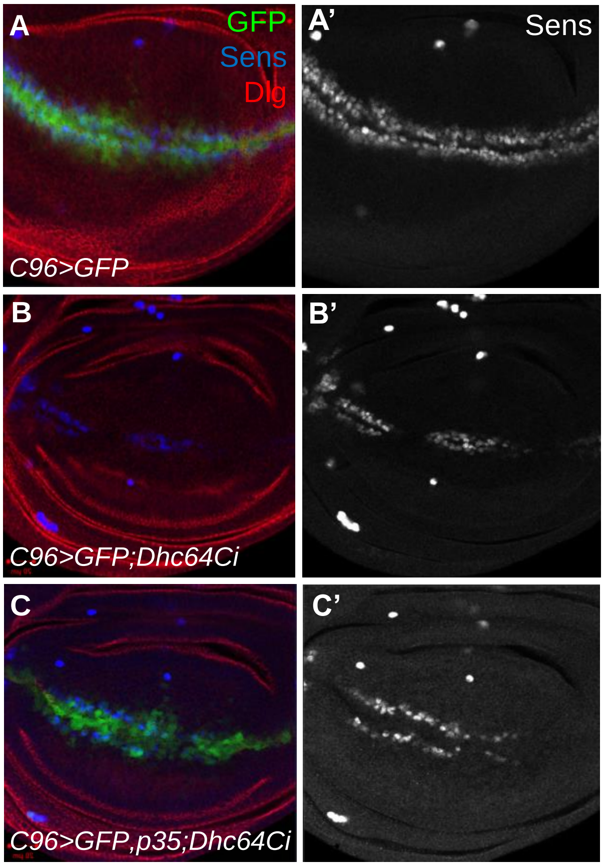

Fig S2. Knockdown of Dhc64C reduces the expression level of Sens, which is partially rescued by coexpression of p35

(A-C) Knockdown of Dhc64C by *C96-Gal4* reduced the level of Sens (B, B') compared to control *C96-Gal4>GFP* (A, A'). Co-expression of *Dhc64C RNAi* with *p35* partially rescued Sens expression (C, C').

Fig S3. (Kim and Orkhon et al)

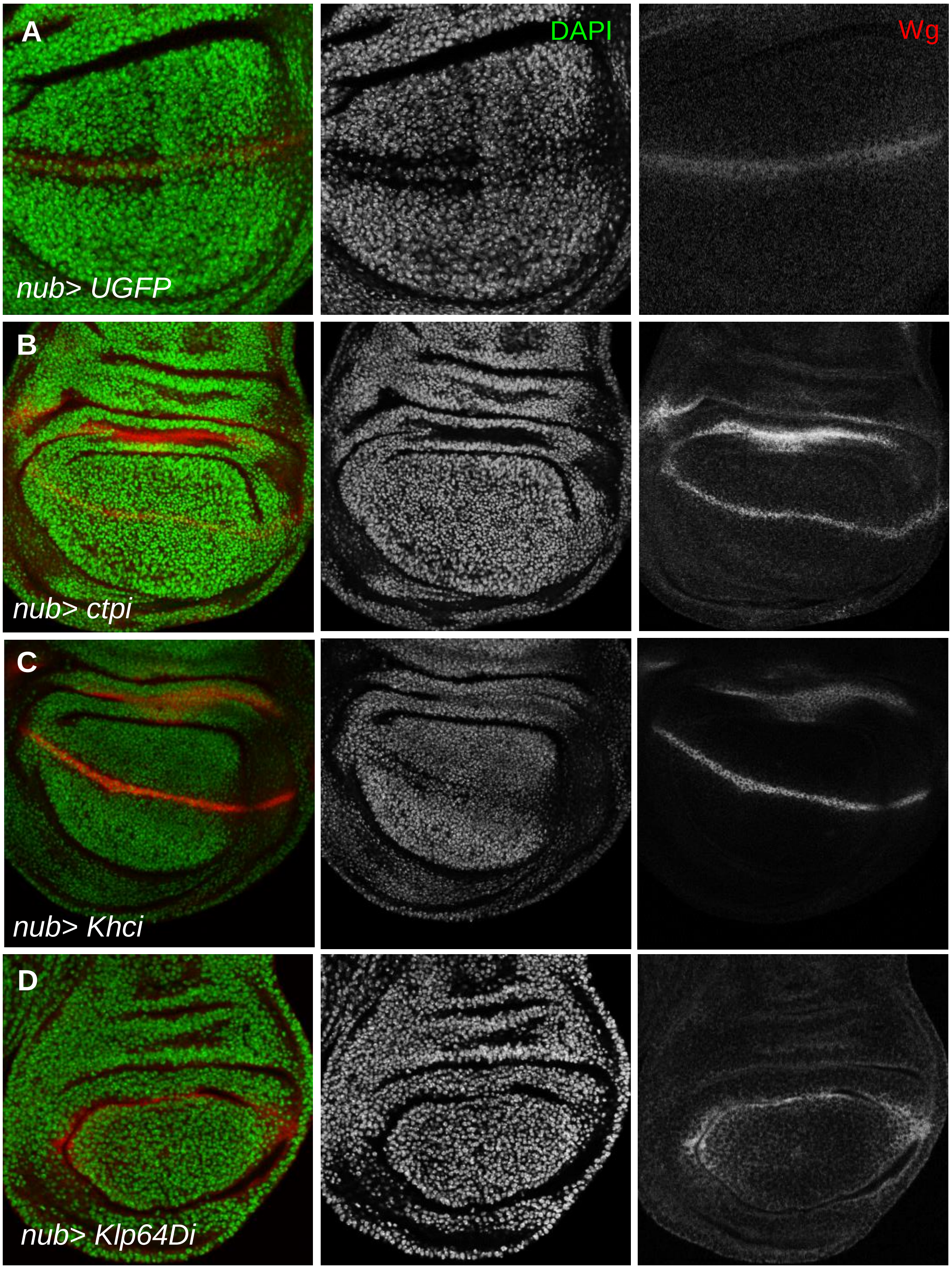

Fig. S3. Subunits in Dynein and Kinesin microtubule motors do not induce overproliferation of Wg-producing cells

Genotypes are written at lower left, and CC3 and Wg are visualized in A’-E’ and A”-E”, respectively. The control nub>GFP wing disc (A) and wing discs with knockdown of Dhc64C (B), ctp (C), Khc (D), Klp64D (E) show differences in the level of Wg and CC3.

Fig S4. (Kim and Orkhon et al)

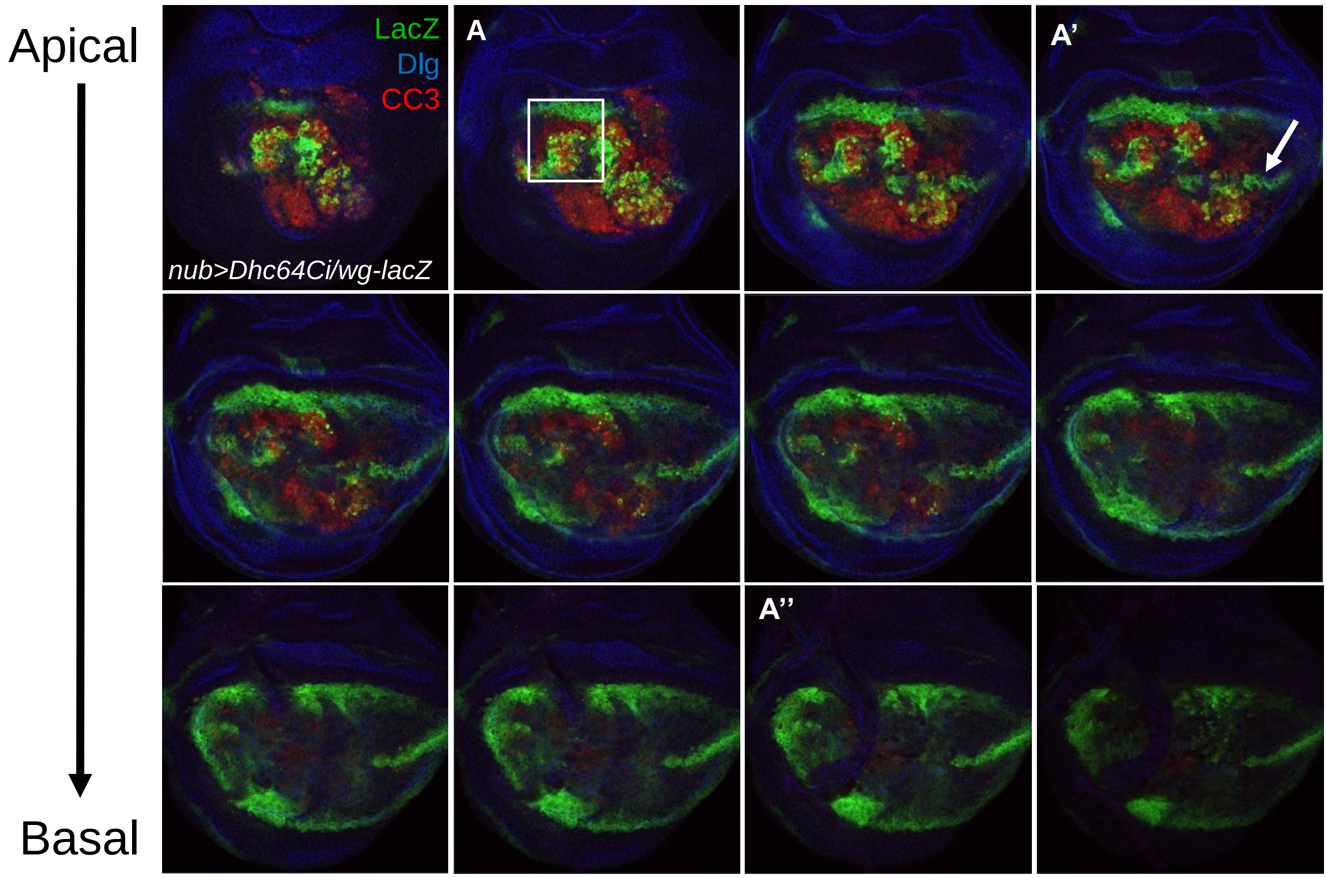

Fig S4. The Wg-LacZ cells in the apical region are apoptotic unlike the Wg-LacZ cells in the basal region

The Z-stack images of a wing disc shown in Fig. 5B, C and D. Wing discs of *nub>Dhc64Ci/wg-lacZ* flies are stained for LacZ, Discs-large (Dlg), and CC3. A , A' and A" are same as Fig. 5C, 5B, and 5D, respectively.
